# LncRNA MCM3AP-AS1 sponges miR-148a to enhance cell invasion and migration in small cell lung cancer

**DOI:** 10.1186/s12885-021-08365-8

**Published:** 2021-07-16

**Authors:** Hua Luo, Yukun Zhang, Guangmei Qin, Bing Jiang, Lili Miao

**Affiliations:** Department of Respiratory and Critical Care Medicine, Yongchuan Hospital of Chongqing Medical University, No.439 Xuanhua Road, Yongchuan District, Chongqing, 402160 P. R. China

**Keywords:** MCM3AP-AS1, Small cell lung cancer, Survival, miR-148a, ROCK1

## Abstract

**Background:**

MCM3AP-AS1 is a recently characterized lncRNA playing an oncogenic role in several cancers. However, its role in lung cancer remains unknown. Here, we aimed to explore the functions of MCM3AP-AS1 in small cell lung cancer (SCLC) and the possible underlying mechanisms.

**Methods:**

MCM3AP-AS1 and ROCK1 levels in SCLC patients were analyzed by qPCR. RNA pull-down and luciferase assays were performed to analyze the interaction between MCM3AP-AS1 and miR-148a. ROCK1 mRNA and protein levels were detected by qPCR and Western blot, respectively. Cell invasion and migration were analyzed by Transwell assays.

**Results:**

MCM3AP-AS1 was upregulated in patients with SCLC, and a high MCM3AP-AS1 level was accompanied by a low survival rate. The binding of MCM3AP-AS1 to miR-148a predicted by bioinformatics analysis was verified by RNA pull-down and luciferase assays. However, MCM3AP-AS1 and miR-148a did not affect each other’s expression. ROCK1 was upregulated in SCLC tissues and positively correlated with MCM3AP-AS1. In SCLC cells, MCM3AP-AS1 overexpression increased ROCK1 and promoted cancer cell invasion and migration, while miR-148a overexpression showed the opposite effects and attenuated the effects of MCM3AP-AS1 overexpression on ROCK1 expression and cell behaviors.

**Conclusions:**

MCM3AP-AS1 sponges miR-148a, thereby increasing SCLC cell invasion and migration via upregulating ROCK1 expression.

**Supplementary Information:**

The online version contains supplementary material available at 10.1186/s12885-021-08365-8.

## Background

Lung cancer, one of the most common cancers, affected 2,093,876 people and caused 1,761,007 deaths worldwide in 2018 [1, 2]. About 15% of lung cancer cases are small cell lung cancer (SCLC), an aggressive form of lung cancer [1]. Smoking is the most common risk factor for SCLC [2]. However, SCLC also affects never-smokers, indicating the critical roles of genetic factors [3]. With the development of novel therapeutic approaches, SCLC patients’ survival rate has been improved during the past decades [1]. However, no significant advance has been achieved in recent years [1].

Studies on the pathogenesis of SCLC have revealed the role of genetic alterations in SCLC tumorigenesis, and targeted therapies have been proposed, designed, tested, and applied [4, 5]. Long non-coding RNAs (lncRNAs) are a class of non-coding RNA molecules that participate in many biological processes by interacting with microRNAs (miRNAs). Extensive studies have uncovered the implication of lncRNAs in the development of cancers. For example, lncRNA DANCR could promote the migration and invasion of gastric cancer cells [6]. LncRNA MIR4435-2HG could induce the tumorigenesis of lung cancer [7]. MCM3AP-AS1 is a recently characterized lncRNA that plays an oncogenic role in several cancers [8, 9]. For instance, MCM3AP-AS1 promotes cancer cell growth in hepatocellular carcinoma by regulating the miR-194-5p/FOXA1 pathway [8]. MCM3AP-AS1 induces angiogenesis in glioblastoma by regulating the miR-211/KLF5/AGGF1 axis [9]. A recent study revealed that YY1-mediated MCM3AP-AS1 overexpression could accelerate tumorigenesis in lung cancer by regulating the miR-340-5p/KPNA4 pathway [10]. However, the potential mechanisms by which MCM3AP-AS1 promotes tumorigenesis in SCLC are not fully elucidated. A better understanding of the interactions between lncRNAs and other molecular players may provide novel insights into the development of anti-cancer therapies [11]. Our bioinformatics analysis showed that miR-148a might bind to MCM3AP-AS1. Previous studies have found that miR-148a has an anti-cancer effect in lung cancer by inhibiting cancer metastasis [12]. Rho-associated coiled-coil-containing protein kinase 1 (ROCK1), a downstream target of miR-148a, has been found oncogenic in many cancers by regulating cancer angiogenesis and metastasis [13, 14]. Therefore, the present study aimed to investigate the role of MCM3AP-AS1 miR-148a in SCLC and whether it functions by regulating the miR-148a/ROCK1 axis.

## Methods

### Patients, treatment, and follow-up

A total of 105 SCLC patients were admitted to Yongchuan Hospital of Chongqing Medical University between April 2010 and April 2014. Among them, 60 patients (42 males and 18 females, 40 to 69 years old, mean age 54.1 ± 6.6 years old) who were newly diagnosed and histopathologically confirmed to have SCLC and had not been previously treated were enrolled in the study. Patients were excluded if they 1) were transferred from other hospitals, 2) had recurrent SCLC, 3) treated previously, and 4) had other multiple clinical disorders. The study was approved by the Ethics Committee of our hospital and was conducted in accordance with the Declaration of Helsinki. The written informed consent was obtained from all patients.

The 60 patients included 15, 20, and 25 cases at AJCC clinical stage II, III, and IV. These patients were treated with surgery only, surgery in combination with chemotherapy or radiotherapy, chemotherapy only, or radiotherapy only based on their conditions and followed up monthly for 5 years since admission via telephone or outpatient visit. Patients who died of causes unrelated to SCLC during the follow up were excluded.

### Specimen collection and SCLC cell model

MRI-guided biopsy was performed to collect both SCLC (tumor) and adjacent non-tumor lung tissues from each patient. Histopathological exams were performed to confirm all tissue samples. Human SCLC cell line SHP-77 was purchased from ATCC (USA) and cultured in 90% RPMI-1640 medium mixed with 10% FBS at 37 °C with 5% CO_2._

### Transient transfections

Negative control (NC) miRNA and miR-148a mimic were purchased from Sigma-Aldrich (USA). Vectors expressing MCM3AP-AS1 and ROCK1 were constructed using pcDNA3 vector by Sangon (Shanghai, China). SHP-77 cells at 80% confluence were transfected with 45 nM miRNA or 10 nM vector using lipofectamine 2000 (Sigma-Aldrich). Un-transfected cells were used as the control (C), and empty vector- or NC miRNA-transfected cells were used as the negative control (NC). At 4 h after transfections, cells were harvested and used in subsequent experiments.

### RNA pull-down assay

Biotinylated-miR-148a (Invitrogen) or -NC miRNA (Bio-NC) was co-transfected into cells with MCM3AP-AS1 expression vector. At 48 h of post-transfection, cell lysates were prepared, streptavidin magnetic beads (Invitrogen) were used to pull down biotinylated miRNAs, and MCM3AP-AS1 level in the precipitates was determined by RT-qPCR.

### Luciferase reporter assay

The wild type (WT) and mutant (Mut) MCM3AP-AS1 were cloned into the promoter region of the firefly and renilla luciferase-containing vector pGL3. The constructed vector and miR-148a were transfected into SHP-77 cells. At 24 h of transfection, relative luciferase activities were assessed using the Dual-Luciferase Reporter Assay Kit (Promega).

### RNA extraction and digestion

Total RNAs in tissue samples (0.03 g per sample) or SHP-77 cells (3 × 10^5^ cells per transfection group) were extracted using RNAzol (Sigma-Aldrich) following the manufacturer’s instructions, treated with DNase I to remove genomic DNA, and precipitated using 75% ethanol to harvest miRNAs.

### qPCR

Tetro Reverse Transcriptase (Bioline) was used to perform all reverse transcriptions. After the addition of poly (A) and miRNA reverse transcription, qPCR assays were performed using the primers MCM3AP-AS1forward CTGCTAATGGCAACACTGA and reverse AGGTGCTGTCTGGTGGAGA, GAPDH forward CAGGAGGCATTGC TGATGAT and reverse GAAGGCTGGGGCTCATTT, ROCK1 forward CCTGTAAC CCAAGGAGATGT and reverse CACAATTGGCAGGAAAGTG, miR-148a forward AAAGTTCTGAGACACTCCG and miR-148a reverse as well as U6 forward and reverse primers. The latter 3 primers were provided in the following kits. MCM3AP-AS1 and ROCK1 mRNA levels were measured using QuantiTect SYBR Green PCR Kit (Qiagen) and normalized to GAPDH. The mature miR-148a level was measured using All-in-One™ miRNA qRT-PCR Detection Kit (Genecopoeia) and normalized to U6. Each PCR reaction was repeated 3 times, and the Ct value was processed using the 2^-ΔΔCT^ method.

### Cell invasion and migration analysis

SHP-77 cells were harvested at 48 h of post-transfection, and cell invasion and migration abilities were analyzed by Transwell assays. Before invasion assays, the membranes were coated with Matrigel (200 μg/ml, Millipore, USA) at 37 °C for 8 h. In brief, cells were prepared as single cell suspensions at 4 × 10^4^ cells per ml of serum-free RPMI-1640 medium. The cell suspensions were loaded onto the upper Transwell chamber (0.1 ml per well), and the lower chamber was filled with 80% RPMI-1640 medium plus 20% FBS. The Transwell plates were incubated at 37 °C for 15 h. Cells in the upper membranes were removed, and the migrated/invaded cells in the lower membrane surfaces were stained with 0.1% crystal violet (Sigma-Aldrich, USA) at room temperature for 15 min and counted under a light microscope.

### Western blot

SHP-77 cells were harvested at 48 h of post-transfection, and ROCK1 expression was detected by Western blot. In brief, total proteins from 10^5^ SHP-77 cells were extracted using 1 ml RIPA solution (Sigma-Aldrich) and quantified using BCA assay (Sigma-Aldrich). The same amounts of proteins were separated on 12% SDS-PAGE gels after denatured in boiling water for 15 min and transferred onto PVDF membranes. After blocked with 5% fat-free milk at 24 °C for 1 h, the membranes were incubated first with primary antibodies against GAPDH (1:1600, ab22555, Abcam) and ROCK1 (1:1600, ab97592, Abcam) at 4 °C for 12 h, then with HRP-labeled goat secondary antibody (IgG) (1:2000; ab6721; Abcam) at 24 °C for 1 h, and last with ECL Western Blotting Substrate (ab65623, Abcam) to develop signals. The signals were processed using Image J v1.46 software.

### Statistical analyses

The mean value of 3 replicates was calculated and used for statistical analyses. Differences between non-tumor and SCLC tissues were compared using the paired t test. Differences among multiple cell groups were analyzed using one-way ANOVA followed by the Tukey test. *P* < 0.05 was statistically significant.

The 60 patients were grouped into low and high MCM3AP-AS1 level groups with the median MCM3AP-AS1 level in SCLC as the cutoff. Their survival curves were plotted and compared by log-rank test. The correlation of the survival rate and MCM3AP-AS1 level in these two groups were analyzed using Pearson’s correlation coefficient.

## Results

### Upregulated MCM3AP-AS1 in SCLC predicted poor survival

**Fig. 1:**
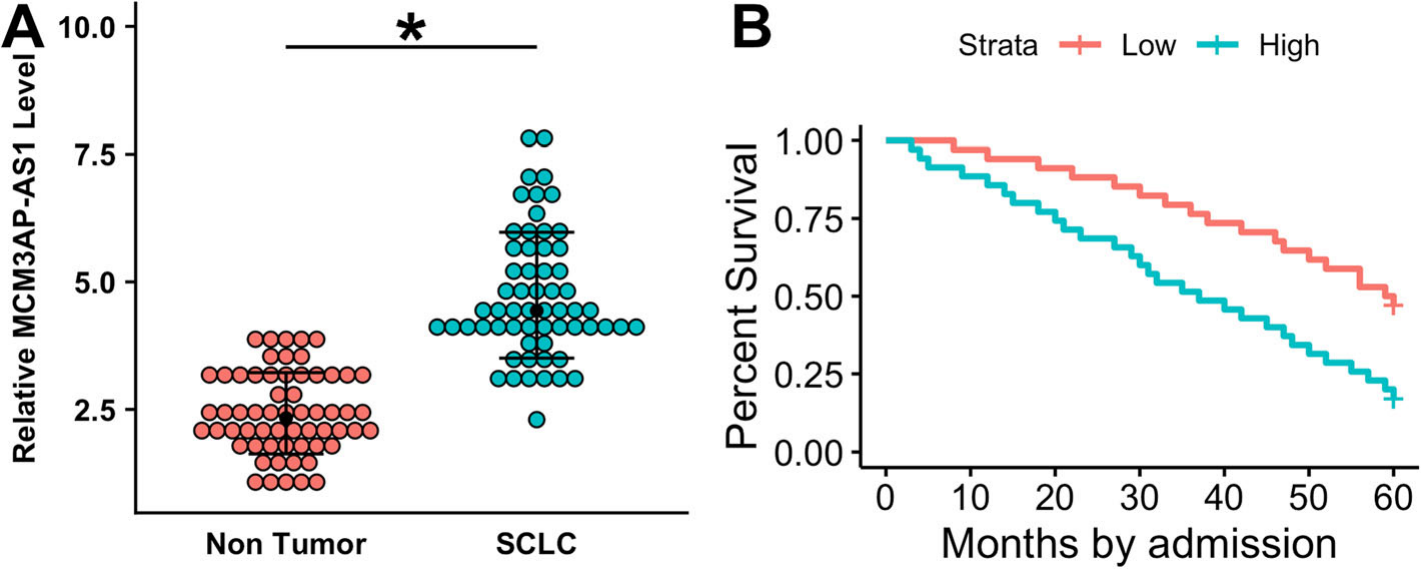
Upregulated MCM3AP-AS1 in SCLC predicted poor survival. The differential expression of MCM3AP-AS1 in SCLC and non-tumor tissues was analyzed by qPCR and paired t test (**A**). To perform survival analysis, the 60 patients were grouped into low and high MCM3AP-AS1 level groups with the median expression level of MCM3AP-AS1 in SCLC as the cutoff value. K-M plotter and log-rank test were used to plot and compare the survival curves (**B**). The mean values of 3 biological replicates were presented. *, *p* < 0.05

The differential expression of MCM3AP-AS1 in SCLC and non-tumor tissues was analyzed by qPCR and paired t test. The results revealed that MCM3AP-AS1 level in SCLC tissues was significantly higher than in non-tumor tissues (Fig. 1A, *p* < 0.05). Survival curve analysis showed that the overall survival rate was significantly lower for patients in the high MCM3AP-AS1 level group than patients in the low MCM3AP-AS1 level group (Fig. 1B).

### MCM3AP-AS1 might bind to miR-148a, while MCM3AP-AS1 and miR-148a did not affect each other’s expression

**Fig. 2:**
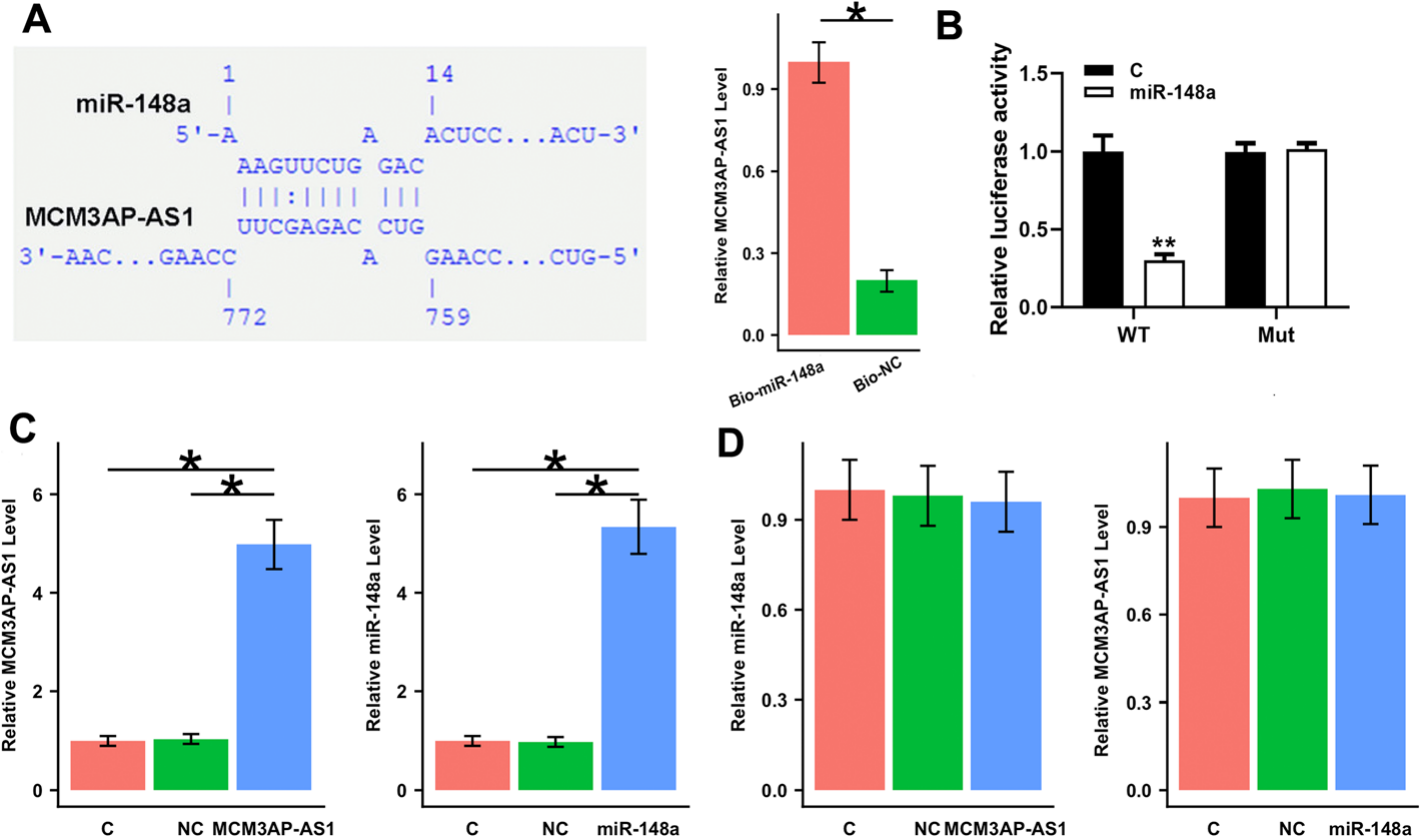
MCM3AP-AS1 might bind to miR-148a, while MCM3AP-AS1 and miR-148a did not affect each other’s expression. The interaction between MCM3AP-AS1 and miR-148a was predicted using IntaRNA (http://rna.informatik.uni-freiburg.de/IntaRNA/Input.jsp). It was observed that miR-148a could form base pairing with MCM3AP-AS1 (**A**, left). The direct interaction between miR-148a and MCM3AP-AS1 was confirmed by RNA pull-down assay (**A**, right) and luciferase reporter assay (**B**). To further analyze the interaction between MCM3AP-AS1 and miR-148a, SHP-77 cells were transfected with MCM3AP-AS1 expression vector or miR-148a mimic. The overexpression of MCM3AP-AS1 and miR-148a was confirmed by qPCR (**C**). The interaction between MCM3AP-AS1 and miR-148a was analyzed by qPCR (**D**). The mean values of 3 biological replicates were presented. *, *p* < 0.05. C: control; NC: negative control; WT: wild type; Mut: mutant type

The prediction for interactions between MCM3AP-AS1 and miR-148a using IntaRNA (http://rna.informatik.uni-freiburg.de/IntaRNA/Input.jsp) showed that miR-148a could form base pairing with MCM3AP-AS1 (Fig. 2A, left). RNA pull-down assay showed that miR-148a could directly interact with MCM3AP-AS1 in cells (Fig. 2A, right, *p* < 0.05). Luciferase assay showed that miR-148a remarkably reduced the luciferase activity in SHP-77 cells transfected with wildtype MCM3AP-AS1 but had no effect on the luciferase activity in SHP-77 cells transfected with mutant MCM3AP-AS1 (Fig. 2B, *p* < 0.05).

qPCR confirmed enhanced MCM3AP-AS1 level in SHP-77 cells transfected with MCM3AP-AS1 expression vector and miR-148a level in SHP-77 cells transfected with miR-148a mimic (Fig. 2C, *p* < 0.05). However, overexpression of MCM3AP-AS1 and miR-148a did not affect each other’s expression (Fig. 2D).

### MCM3AP-AS1 was positively correlated with ROCK1 in SCLC

**Fig. 3:**
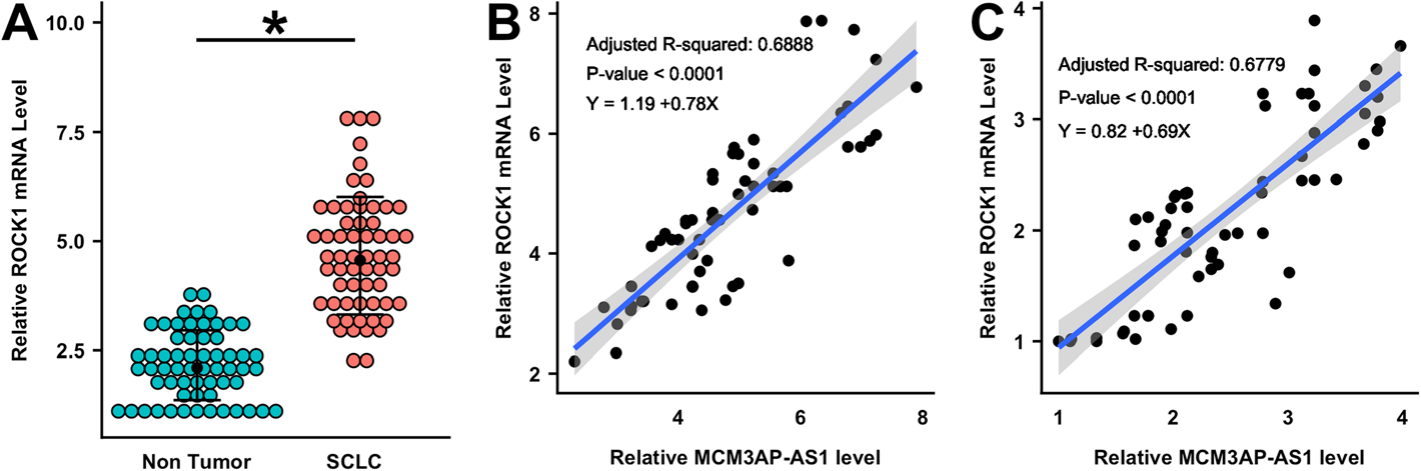
MCM3AP-AS1 positively correlated with ROCK1 in SCLC. ROCK1 is a direct target of miR-148a. The differential expression of ROCK1 mRNA in SCLC and non-tumor tissues was analyzed by qPCR and paired t test (**A**). Correlations between MCM3AP-AS1 and ROCK1 mRNA across SCLC (**B**) and non-tumor (**C**) tissues were analyzed by Pearson correlation coefficient. The mean values of 3 biological replicates were presented. *, *p* < 0.05

ROCK1 is a direct target of miR-148a. The differential expression of ROCK1 in SCLC and non-tumor tissues was analyzed by performing qPCR and paired t test. The results revealed that ROCK1 expression was significantly higher in SCLC tissues than in non-tumor tissues (Fig. 3A, *p* < 0.05). Correlations between MCM3AP-AS1 and ROCK1 across SCLC and non-tumor tissues were analyzed, and the results indicated that MCM3AP-AS1 and ROCK1 levels were positively correlated in both SCLC (Fig. 3B) and non-tumor (Fig. 3C) tissues.

### MCM3AP-AS1 upregulated ROCK1 via miR-148a

**Fig. 4:**
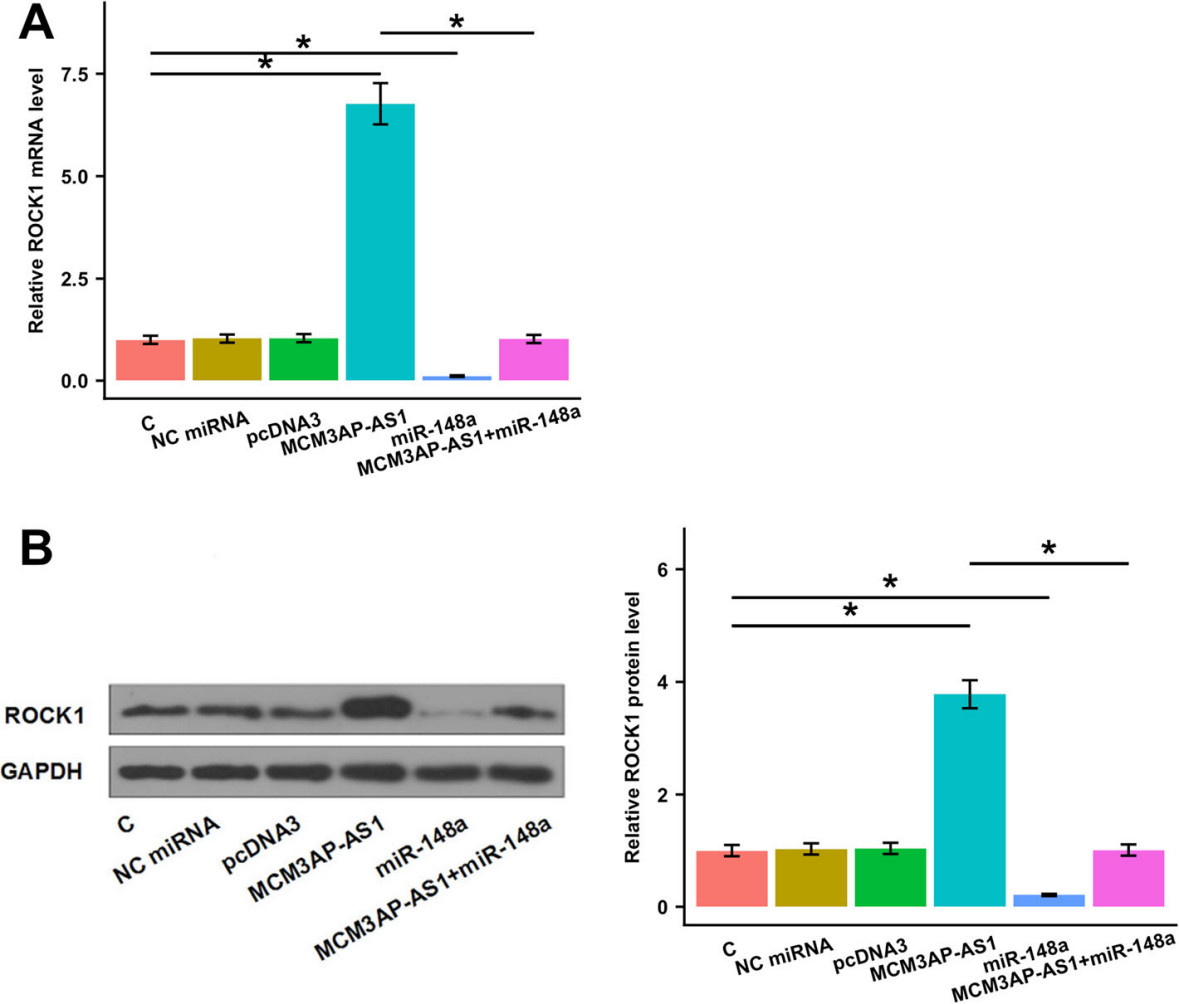
MCM3AP-AS1 upregulated ROCK1 via miR-148a. The effects of MCM3AP-AS1 and miR-148a overexpression on ROCK1 expression at mRNA (**A**) and protein (**B**) levels were analyzed by qPCR and Western blot, respectively. The mean values of 3 biological replicates were presented. *, *p* < 0.05. C: control; NC: negative control

Compared to the control group, MCM3AP-AS1 overexpression remarkably upregulated ROCK1 at both mRNA (Fig. 4A, *p* < 0.05) and protein (Fig. 4B, *p* < 0.05) levels. By contrast, miR-148a overexpression obviously decreased ROCK1 expression level (Fig. 4A-B, *p* < 0.05). Besides, experiments on cells co-transfected with miR-148a mimic and MCM3AP-AS1 expression vector showed that miR-148a overexpression attenuated the effects of MCM3AP-AS1 overexpression on ROCK1 level (Fig. 4A-B, *p* < 0.05).

### MCM3AP-AS1 promoted SCLC cell invasion and migration via ROCK1 and miR-148a

**Fig. 5:**
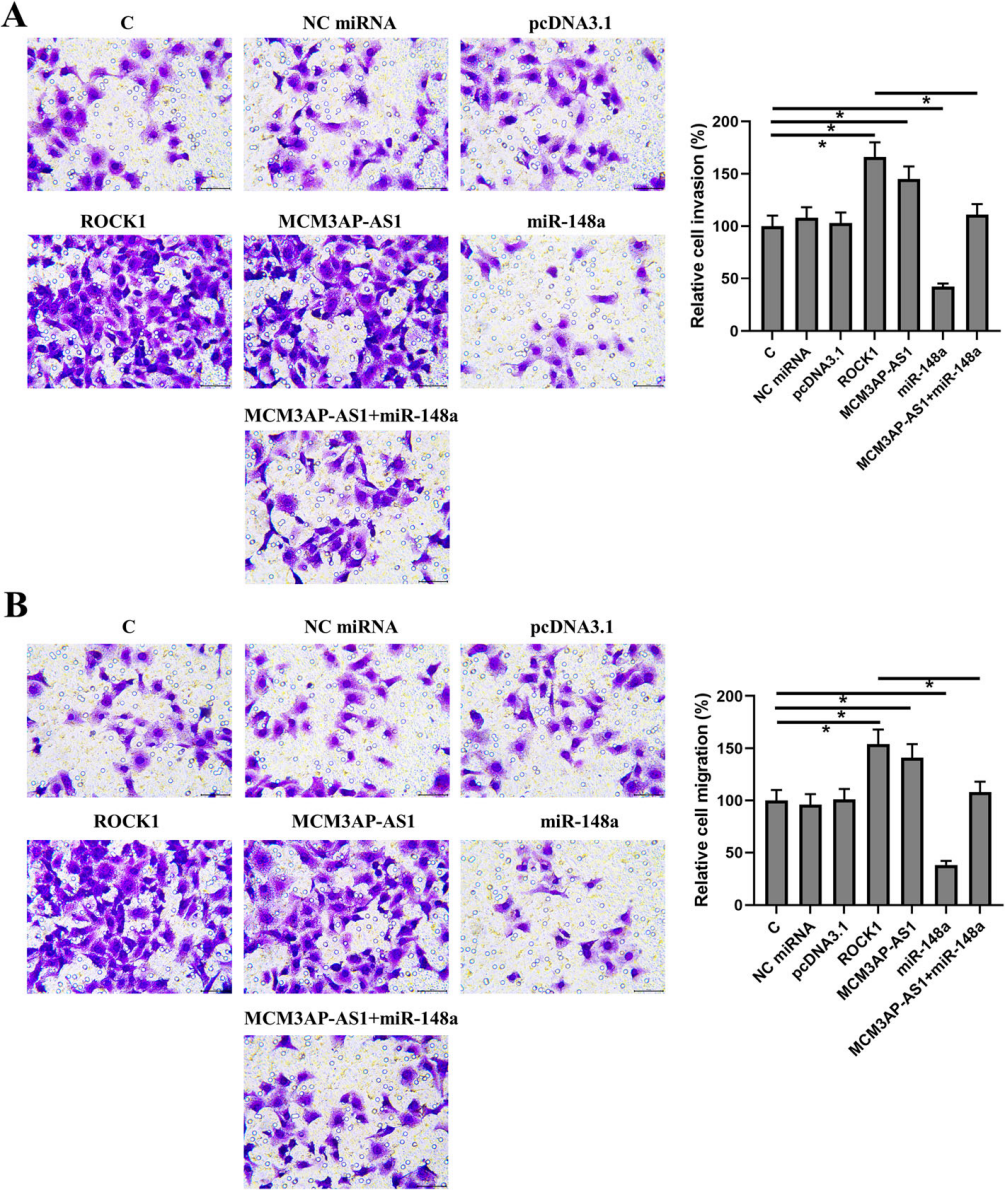
MCM3AP-AS1 promoted SCLC cell invasion and migration via ROCK1 and miR-148a. The effects of overexpression of MCM3AP-AS1, miR-148a, and ROCK1 on the invasion (**A**) and migration (**B**) of SHP-77 cells were analyzed by Transwell invasion and migration assays, respectively. The mean values of 3 biological replicates were presented. *, *p* < 0.05. C: control; NC: negative control

The effects of overexpression of MCM3AP-AS1, miR-148a, and ROCK1 on the invasion and migration of SHP-77 cells were analyzed by Transwell invasion and migration assays, respectively. ROCK1 overexpression was confirmed by Western blot (Supplemental Fig. 1, *p* < 0.05). Compared to the control group, overexpression of MCM3AP-AS1 and ROCK1 markedly increased cell invasion and migration rates (Fig. 5A-B, *p* < 0.05). However, experiments on cells co-transfected with miR-148a mimic and MCM3AP-AS1 expression vector showed that miR-148a overexpression significantly decreased cell invasion and migration rates and attenuated the effects of MCM3AP-AS1 overexpression on cell invasion and migration (Fig. 5A-B, *p* < 0.05).

## Discussion

**Fig. 6:**
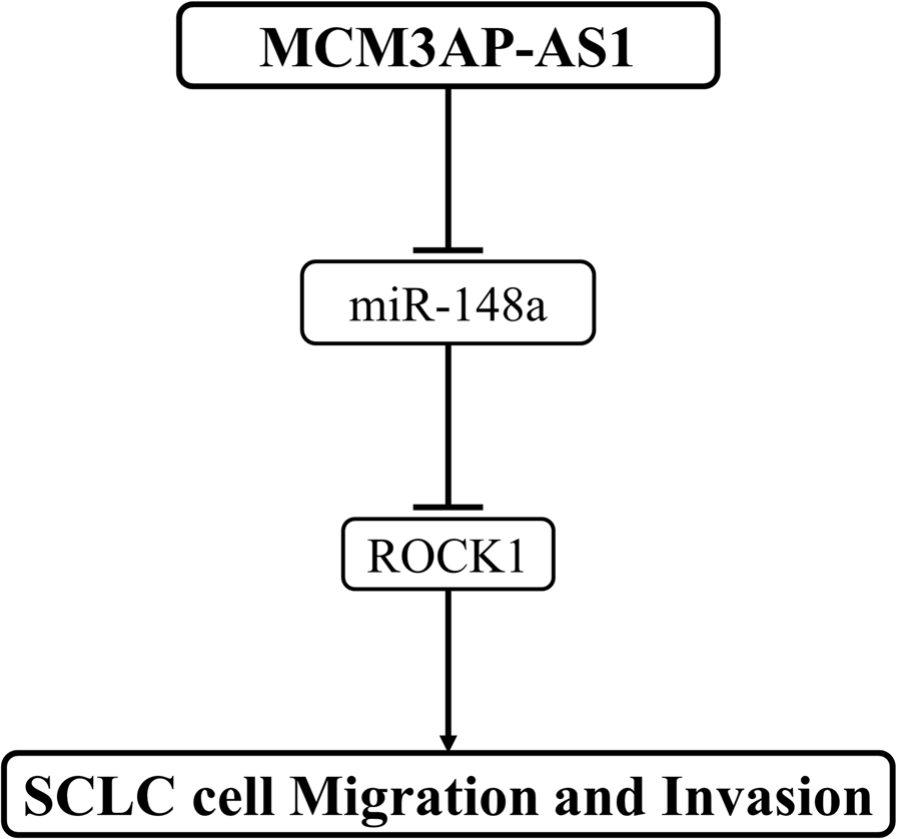
The schematic diagram of the proposed mechanism. MCM3AP-AS1 promotes SCLC cell migration and invasion by regulating the miR-148a/ROCK1 axis

The function and expression pattern of MCM3AP-AS1 in SCLC were investigated in this study. The results showed that MCM3AP-AS1 was significantly upregulated in SCLC, and high MCM3AP-AS1 level predicted poor survival of SCLC patients. In addition, MCM3AP-AS1 might upregulate ROCK1 by sponging miR-148a (Fig. 6).

LncRNAs have been recognized as critical regulators in the development of lung cancer. For instance, lncRNA MIR4435-2HG promotes lung cancer progression, while TFPI2AS1 represses lung cancer cell proliferation and migration [7, 15]. Recently, MCM3AP-AS1 was recognized as an oncogenic lncRNA in several cancers. In hepatocellular carcinoma, MCM3AP-AS1 is upregulated and inhibits cancer growth by regulating the miR-194-5p/FOXA1 axis [8]. In glioblastoma multiforme, MCM3AP-AS1 is also upregulated and interacts with the miR-211/KLF5/AGGF1 axis to promote tumor angiogenesis [16]. Although the general oncogenic function of MCM3AP-AS1 has been reported in lung cancer [13], its specific role in SCLC remains unknown. This present revealed MCM3AP-AS1 upregulation in SCLC. Our in vitro cell experiments showed that MCM3AP-AS1 overexpression markedly enhanced SHP-77 cell invasion and migration, suggesting that MCM3AP-AS1 is an oncogenic lncRNA in SCLC. Five-year follow-up revealed the prognostic value of MCM3AP-AS1 for SCLC patients: high MCM3AP-AS1 level was significantly correlated with the poor survival of SCLC patients. However, more clinical trials are needed to further verify this finding and evaluate its reliability and accuracy.

The tumor suppressive roles of miR-148a have been investigated in many cancers, including non-SCLC (NSCLC), another form of lung cancer [17, 18]. In NSCLC, miR-148a inhibits cancer metastasis, and decreased serum miR-148a has been considered a screening marker of NSCLC [12]. However, the function of miR-148a in SCLC has not been well-studied. This study revealed the tumor suppressive role of miR-148a in SCLC by inhibiting cancer cell invasion and migration. It is known that miR-148a can directly target ROCK1 in gastric cancer and NSCLC [17, 19]. Consistently, this study revealed that miR-148a overexpression reduced ROCK1 expression, indicating that miR-148a may target ROCK1 in SCLC. ROCK1, as an effector of the small GTPase RhoA, is usually upregulated in different cancers and can increase cancer cell motility to promote tumor invasion and metastasis [20]. In lung cancer, ROCK1 is involved in multiple pathologic processes, such as cancer cell proliferation, migration, and invasion, thereby promoting lung cancer tumorigenesis [21]. Similarly, our results showed the enhancing effects of ROCK1 on SCLC cell invasion and migration. Taken together, miR-148a might serve as a potential drug candidate to inhibit tumor metastasis in SCLC.

MiRNAs mainly function as gene expression regulators by cleaving mRNAs or inhibiting mRNA translation [22]. This study found that miR-148a might bind to MCM3AP-AS1 but had no regulatory role on MCM3AP-AS1 expression. It is known that lncRNAs can sponge miRNAs to attenuate their effects on downstream genes [23]. Combined with the observation of upregulated ROCK1 after MCM3AP-AS1 overexpression, we speculated that MCM3AP-AS1 might sponge miR-148a to upregulate ROCK1. Previous studies have found that multiple lncRNAs can affect cancer development by regulating ROCK1 via sponging the corresponding miRNAs, consistent with our findings. For example, lncRNA DANCR induces cervical cancer progression by regulating the miR-335-5p/ROCK1 axis [24]. LncRNA SNHG1 promotes osteosarcoma development by regulating the miR-101-3p/ROCK1 axis [25]. It is worth noting that the present study is limited by the small sample size of SCLC patients and the lack of animal experiments. Our findings need to be further verified using more SCLC cases and animal experiments.

## Conclusions

The study revealed that MCM3AP-AS1 was significantly upregulated in SCLC. In addition, MCM3AP-AS1 enhanced ROCK1 expression via sponging miR-148a and promoted SCLC cell invasion and migration by regulating the miR-148a/ROCK1 axis. Therefore, MCM3AP-AS1 could serve as a potential therapeutic target for SCLC.

## Supplementary Information

**Additional file 1: Supplemental Figure 1.** Confirmation of ROCK1 overexpression by Western blot. Overexpression of ROCK1 in SHP-77 cells was confirmed by Western blot. The mean values of 3 biological replicates were presented. *, *p* < 0.05. C: control; NC: negative control.

## Data Availability

The datasets generated and/or analyzed during the current study are available at https://pan.baidu.com/s/13ieC350zUw0kGOeqp%2D%2DhYw. The code is gheu.

## Abbreviations

HCC: Hepatocellular carcinoma
SCLC: Small cell lung cancer
lncRNA: Long non-coding RNA
NC: Negative control
