# Supplementary figures and images for: LncRNA MCM3AP-AS1 sponges miR-148a to enhance cell invasion and migration in small cell lung cancer
Source: BMC Cancer. 2021 Jul 16;21:820. doi: 10.1186/s12885-021-08365-8 (PMC8283830; doi:10.1186/s12885-021-08365-8)

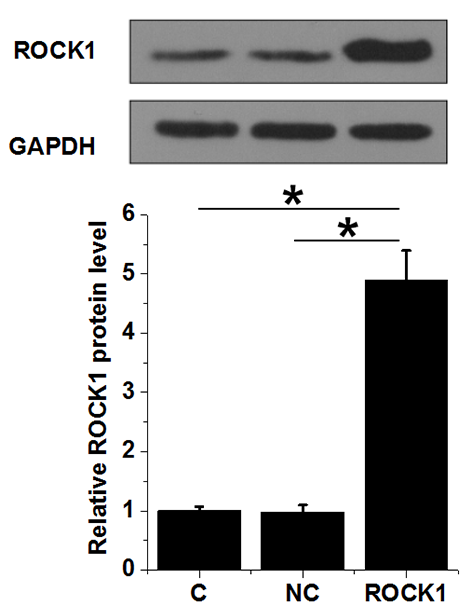

Supplement: Supplementary file 1 — Additional file 1: Supplemental Figure 1. Confirmation of ROCK1 overexpression by Western blot. Overexpression of ROCK1 in SHP-77 cells was confirmed by Western blot. The mean values of 3 biological replicates were presented. *, p < 0.05. C: control; NC: negative control. [file 12885_2021_8365_MOESM1_ESM.tif]
